# The use of 3D surface scanning for the measurement and assessment of the human foot

**DOI:** 10.1186/1757-1146-3-19

**Published:** 2010-09-05

**Authors:** Scott Telfer, James Woodburn

**Affiliations:** 1 School of Health, Glasgow Caledonian University, Cowcaddens Road, Glasgow, G4 0BA, UK

## Abstract

**Background:**

A number of surface scanning systems with the ability to quickly and easily obtain 3D digital representations of the foot are now commercially available. This review aims to present a summary of the reported use of these technologies in footwear development, the design of customised orthotics, and investigations for other ergonomic purposes related to the foot.

**Methods:**

The PubMed and ScienceDirect databases were searched. Reference lists and experts in the field were also consulted to identify additional articles. Studies in English which had 3D surface scanning of the foot as an integral element of their protocol were included in the review.

**Results:**

Thirty-eight articles meeting the search criteria were included. Advantages and disadvantages of using 3D surface scanning systems are highlighted. A meta-analysis of studies using scanners to investigate the changes in foot dimensions during varying levels of weight bearing was carried out.

**Conclusions:**

Modern 3D surface scanning systems can obtain accurate and repeatable digital representations of the foot shape and have been successfully used in medical, ergonomic and footwear development applications. The increasing affordability of these systems presents opportunities for researchers investigating the foot and for manufacturers of foot related apparel and devices, particularly those interested in producing items that are customised to the individual. Suggestions are made for future areas of research and for the standardization of the protocols used to produce foot scans.

## Background

The use of 3D surface scanning technologies to produce digitised representations of parts of the human anatomy has the potential to help change the way a wide range of products are designed and fabricated [1]. Until recently, the anthropometric databases that are used by designers and manufacturers to guide the ergonomic form of their products have primarily been based on 1D and 2D measurements, for example leg length or waist girth [2]. This approach results in approximations being made when designing to body areas for which an easily defined measurement is not available. Databases that draw upon 3D scans can offer far more detailed information on the contours of the body and potentially provide an insight into changes in anthropometric measurements associated with dynamic movement. Indeed, initiatives such as the CAESAR study (Civilian American and European Surface Anthropometry Resource) [3] have been carried out with the aim of collecting this type of information. 3D surface scanning has the potential to play an important role in the development of customised products, i.e. devices and apparel that are designed for the individual using their precise anthropometric measurements [4,5].

In the case of the foot, quantitative description of its shape is important for a number of different applications relating to the ergonomic design of footwear, foot orthotics and insoles, and for research into and clinical assessment of foot deformities, such as those associated with rheumatoid arthritis [6-9]. Additionally, because the foot is a flexible and complex structure, a better understanding of how its shape changes in different situations, for example in the different loading phases of the gait cycle, may lead to improvements in the overall comfort and functionality of the footwear and devices that are been produced [10].

**Figure 1:**
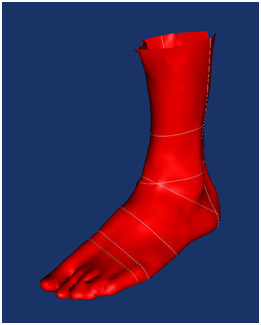
**Scan of the foot (taken using Easy-Foot-Scan from OrthoBaltic (Kaunas, Lithuania)**.

There are now a number of surface scanning systems (costing between €5,000 and €30,000) available which can scan the plantar surface of the foot or the leg and foot (see Figure 1). This produces a 3D representation of its shape that can be viewed and analysed on a computer. Software programs which allow these 3D models to be used as the basis for shoe or foot orthotic design and integrate with computer controlled manufacturing systems are now widely available. This has meant that a number of footwear companies are now using integrated customisation systems to produce customer-specific shoes [11], and similarly there are now manufacturers providing customised foot orthotics that are based directly upon a scan of the patient's foot shape [12,13]. While the current volumes of these goods are relatively low, it is thought that as the price and lead times for these items fall their share of the market will increase [14].

The aim of this review is to summarise the ways 3D scanning technologies have been used in research relating to the design of customised foot orthotics and footwear, and for the anthropometric measurement and assessment of the foot.

## Methods

### Search strategy

Initial searches for this review were carried out in March/April 2010 in the PubMed, and ScienceDirect databases. Reference lists were examined and experts in the field consulted for additional related articles. Inclusion criteria were that 3D surface scanning of the foot was an integral part of the study protocol, the article was written in English and that it was from a peer reviewed publication or conference proceedings. From the original database searches 141 unique articles were identified, 19 of which met the inclusion criteria (table 1). An additional 18 articles meeting the inclusion criteria were identified from other sources.

**Table 1:** Search strategy

PubMed			
**Search term**	**Results**	**Relevant**	**Reference numbers**

3D foot scan*	3	3	[8,50,53]
Foot digitizer	13	1	[23]
Foot digitiser	2	0	
Foot surface scan*	2	1	[45]
Foot shape scan*	62	6	[6,19-21,23,32]
Foot shape digiti*	14	2	[23,25]
Foot weight bearing scan	41	0	
Foot anthro* scan	0	0	
			

**ScienceDirect**			

3D foot scan*	20	4	[5,16,17,49]
Foot digitizer	5	0	
Foot digitiser	0	0	
Foot surface scan*	64	5	[17,33,40,49,53]
Foot shape scan*	20	3	[17,40,49]
Foot shape digiti*	9	1	[12]
Foot weight bearing scan	5	0	
Foot anthro* scan	7	2	[5,16]

## Results

### Scanning/digitising

The latest technologies available to produce 3D representations of the foot can be split into two loose categories: scanners and digitisers. Scanning is a process where 3D images are converted to digital form using optical or video equipment; and digitising involves the 3D shape having its features traced and stored as digital codes on a computer. Scanning differs from digitising in that entire pages of data are captured at once, whereas with digitising discreet points are entered one at a time. In many modern systems the line between the two technologies has become somewhat blurred and for the purposes of this review no particular distinction has been drawn between foot scanners using the differing approaches. Additionally, systems that can capture the 3D shape of the foot during motion have recently become available, meaning that foot scanners can also be divided into dynamic and static types. The latest research using both types is covered in this review.

### Foot measurements

#### Linear measurements

The most common foot measurements taken are those made by shoe retailers who use the length of the foot in order to sell their customers the closest fitting shoe from their stocked range of sizes. These sizes are defined by various national and continental sizing systems [15,16]. Retailers may use a Brannock device which, as well as length, can also measure width and arc length of the foot. Depending on the retailer/country however, a simpler device which measures only length may be used. In general, for reasons of economy footwear manufacturers tend only to provide a standard width and height associated with each shoe size.

There is some variation as to the approaches and anatomical landmarks are used to define foot length. Typically it is taken as being from the pternion to the tip of the second toe but on the Brannock device it is defined by using an axis orientation by a line joining the pternion and a point 38.1 mm lateral to the medial edge of the first metatarsal head. There are some limitations with many of the standard measurement conventions, mainly relating to when the foot being measured features a deformity such as hallux valgus [16].

An international standard, ISO 20685, has been produced with the aim of ensuring that measurements taken using 3D scanning systems are comparable with those taken using traditional methods and can be used in anthropometric databases. This standard is limited to measurements of foot length and breadth, and requires that the maximum mean difference between the traditional and 3D scanning derived values is 2 mm. Most modern foot scanners however claim to have sub-millimetre accuracy.

Methodologies for automatically generating foot measurement data from 3D digitisations have been produced [16,17]. After correction for systematic errors, Witana et al [16] were able to show that there were no significant differences between the automatically generated measurements and those taken manually. Prior to the measurements being generated, the foot scans need to be aligned in order for the measurements to be repeatable, and research has been carried out investigating the variation resulting from the different processes that can be used to achieve this [18]. The findings from this work suggested that the measurements are sensitive to the alignment process used and the authors recommended that dimensions should be based on anatomical landmarks that are independent of the registration process.

Investigations have been carried out using 3D scanning in an attempt to provide information relevant to shoe designers. A large study was carried out by Krauss et al [19] who used the data generated to categorise the foot into different types: voluminous, flat pointed and slender. A similar study by the same group involved the scanning of the feet of 2867 children and again the authors were able to categorise the results into three foot types [20]. Luo et al [21] used 3D scanning to assess the differences in male and female feet and found that men tend to have longer and wider feet than women, in line with results from previous studies that took manual measurements [22].

#### Changes due to weight bearing

A research area where 3D scanners and digitisers have been utilised is in measuring the changes that occur to foot shape between non-weight bearing and weight bearing states.

When loaded, there are a number of anthropometric changes that occur in the foot. Several studies have investigated these changes, the majority simply measuring the differences under varying loading conditions using traditional methods. In recent years 3D scanners have been used for this purpose, either by directly scanning the foot while loaded [23,24], or by scanning casts of it that were taken while weight was applied [25]. Selected results from these studies are summarised and combined in Table 2. It has been suggested that this approach to making the anthropometric measurements can potentially reduce errors resulting from skin displacement and tissue distortion that can occur when using callipers or other measuring tools.

**Table 2:** Selected results from studies using 3D scanners to measure anthropometric changes in the foot under weight bearing conditions.

		Percentage change in parameter			
			
Foot shape parameter	Load	**Houston et al (40 feet) **[38]	**Tsung et al (16 feet) **[39]	**Xiong et al (60 feet) **[40]	Mean
Length	HWB	+1.7%	+2.7%	+1.1% (M 1.1%, F1.1%)	+1.53%
	FWB	+2.2%	+3.4%	+1.4% (M1.3%; F1.5%)	+1.95%
Ball width	HWB	+3.8%	+2.9%	+2.2% (M2.6%; F 1.7%)	+2.85%
	FWB	+4.3%	+6%	+2.6% (M3.1%; F2%)	+3.66%
Heel width	HWB	+4.8%	+5.9%	+1.1% (M1.4%; F0.8%)	+3.04%
	FWB	+4.8%	+8.7%	+1.6% (M1.8%; F1.3%)	+3.68%

Percentage changes relate to baseline measurements taken of the unloaded foot, HWB: half weight bearing; FWB: full weight bearing.

Overall, the combined results from the studies using 3D scanners compare favourably with the previous literature, reinforcing findings from studies using other measuring techniques suggesting that the increases in length and breadth between unloaded and half loaded are greater than those found between half and full weight bearing [26]. Variation in the results between studies could be related to differences in the populations the study sample was drawn from and protocol used to obtain the measurements. For example, Tsung et al took their measurements from a cast of the foot rather than a direct scan, and it is possible that this could have had some effect on foot shape or in the measurement and this could explain why, in particular, the heel width value was found to be so much greater in that study. The amount of variation between studies in the results between studies does suggest that the protocol used to scan the foot and acquire the data has a strong influence on the outcome measurements and that this should be standardised where possible.

#### Girth measurements

Beyond the relatively simple length and width measurements, it is necessary to move back a stage to the fabrication of the shoes themselves to find where more comprehensive data on the overall foot shape are required. Currently, the most common method of manufacturing a shoe - whether customised or mass produced - requires a shoe last, which is the wooden or metal model of the foot around which the materials that form the shoe are shaped [27]. The development of the last requires a number of foot measurements in order for it to accurately represent the individual foot or the average foot shape for a particular shoe size.

At the moment lasts tend to be manufactured by experienced shoemakers. Attempts have been made to modernise the design and manufacture of lasts, using computerised design to replace more variable artisan skills. It is here that 3D scanning technologies provide the opportunity to take an extensive range of measurements from the digitisation of the foot or to use modern fabrication techniques such as rapid prototyping to manufacture a last based directly on the computer model [28,29]. An early attempt was made by Bao et al [30] to define an integrated system for the manufacture of personalised shoe lasts that were to be used in the design of orthopaedic shoes. This was based on the 3D scanning of the foot followed by manufacture of the last using CAM, and this basic approach is still followed in shoe customisation today.

Traditional manufacturers of customised shoes use a tape measure to obtain girth measurements in an attempt to provide a better fit [16]. Using a tape to manually measure girth can be inaccurate as the irregular shape of the foot can mean that the tape is not in contact with the surface the whole way around the foot. However, because shoe last design has been successfully based around these types of measurements for the past century and earlier, researchers using representations of the foot developed from 3D scanning have, with some success, sought to emulate the shape of the tape during girth measurements using various algorithms in order to include the non-contact areas [5,16].

Researchers have used 3D scanners to investigate the quality of fit between foot and shoe. Nacher et al [31] took foot scans of 316 participants as well as their preferences regarding shoe fit and produced a model able to predict fitting level with an accuracy of 65.7%. Witana et al [32] scanned the lasts for four pairs of men's shoes and compared them to the foot scans of a group of males, finding that there were significant differences between the two shapes. The authors also proposed a method of improving footwear fit through matching of 2D outlines of the scans and identifying areas where there could potentially be fit problems. Wang [33] built on this work by scanning a library of 10 shoe lasts and developing a process for choosing the last from the library that would be most suited for the individual based on their ball girth, waist girth and instep girth of their foot.

Luximon and Goonetilleke [6] have argued that the foot shape can be modelled using just length, width, height and a measure of the curvature of the metatarsal-phalangeal joint in order to negate the use of 3D scanners. Using these variables they were able to predict individual foot shape to a mean accuracy of 2.4 mm. While perhaps acceptable for the general populace, this approach is not suitable for those with foot deformities and further research is required to determine how accurate the fit needs to be in order to affect comfort and other biomechanical factors.

### Plantar surface shape measurement

For a number of conditions, customised foot orthotics have been shown to be more effective at reducing pain and redistributing pressure than standard "off the shelf" orthotics [34]. Traditionally, customised foot orthotics are fabricated by taking a plaster cast of the plantar surface of the patient's foot (the negative cast), making a positive plaster cast of the foot by filling the negative cast, and then moulding the orthotic around the positive cast to obtain a high quality fit [35]. The positive cast can be altered either by removing or adding plaster to it so that, for example, the orthotic will take pressure away from certain areas of the foot or provide support to the arch.

**Figure 2:**
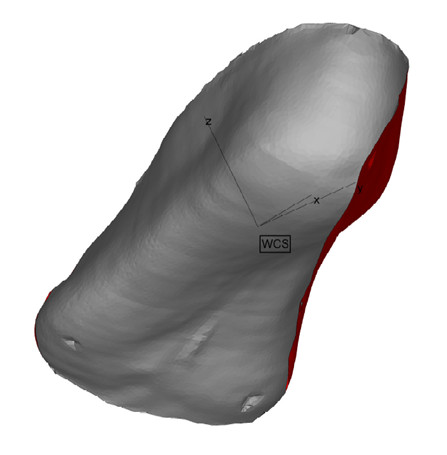
**Scan of negative cast of the foot (3/4 length)**. 1^st ^and fifth metatarsal heads can be seen to be marked on cast. Scan taken using a hand held Cobra 3D scanner (Polhemus, Colchester, VT, USA) and image viewed using MiniMagics .stl viewer (Materialise, Leuven, Belgium).

Modern scanning systems allow the "positive" shape of the foot to be obtained directly, circumventing the need to cast the foot (although some handheld systems do allow a cast of the foot to be scanned (see Figure 2)). A number of software packages (for example Orthomodel from Delcam PLC, Birmingham, UK; and Automated Orthotic Manufacturing System, Sharp Shape, CA, USA) have been developed which have the ability to design foot orthotics based directly on the 3D representations of the foot obtained by surface scanning.

The software, as well as matching the shape of the foot sole, allows the user to alter the shape and thickness of the orthotic in a controlled manner, giving greater design freedom than traditional plaster cast methods. By linking up with computer controlled milling or routing machines that can manufacture the orthotics, this approach reduces the number of steps in the process as well as removing many of the sources of human error.

There are alternative methods of obtaining the shape of the foot. Rather than casting or scanning the foot directly, in recent years an impression foam system has gained popularity [36]. With this method, the patient stands on or has their foot pushed into a low density foam box. The foam collapses under their weight and when they step out of the box there is a fairly close negative of the shape of their foot left in the foam. The box can then either be filled with plaster to obtain a positive cast or a number of companies now take a 3D scan directly from the impression box and use this to guide the machining of the orthotic. This is a fast and inexpensive method of obtaining the shape of the plantar surface of the foot.

#### Comparison of foot orthotics designed using different methods

The literature directly comparing the effects of orthotics made via these different methods is limited. A recent study by Pallari et al [37] compared traditionally produced orthotics with those developed from a 3D computer model and fabricated using selective laser sintering found the two sets to be comparable in terms of observed gait, comfort and fit. Efforts have been made to compare orthotics designed using a digitisation method to those produced using the foam impression technique and analysis of the results found that the orthotics produced by the CAD CAM method to be more effective at redistributing pressure away from the forefoot region and supporting the transverse arch [38].

Laughton et al [39] made a comparison between four different methods of obtaining a negative impression of the foot, including two derived from laser scans (one low weight bearing and one partial weight bearing). The results showed that there were significant differences between the measurements obtained using different methods. For example, plaster casting tending to produce results closest the clinical measurements for the forefoot-to-rearfoot relationship, but the partial weight bearing laser scans showing the closest correlation for forefoot width. The study suffered from difficulties in positioning the foot on the scanner for the non-weight bearing scan, a factor which the authors acknowledged may have affected the results for this technique.

Early work by Foulston et al [40] used a basic digitising system to compare the casts taken with the foot in unsupported and corrected positions. This system used electromagnetic technology rather than optical and only recoded a limited number of points on the cast's surface compared to modern scanners. The authors however were able to capture and analyse the differences in the plantar surface shape between the two positions.

#### Cost

In terms of cost, it has been suggested that acquiring a 3D image of the foot using an optical scanner or digitiser and basing the orthotic on it can offer significant savings over traditional plaster cast methods [7]. The cost of taking a plaster cast and preparing it for prescription including materials and clinician time has been estimated at €20-34 (it should be noted that the figures used in this study were only for the Australian market, however it is thought that these could be extrapolated to European and other developed markets for foot orthotics). In comparison, a scanner generated representation of the plantar surface of the foot is estimated to cost €2.25-6.80. Further costs can be incurred with the traditional method if the cast has to be transported to a different location in order for the orthotic to be made, whereas the scan file can simply be emailed.

From the perspective of a clinician prescribing the orthotic, there is therefore a strong financial incentive to move away from traditional casting techniques. However, the initial capital outlay for a 3D scanner or digitiser is much higher than the amount required for plaster casting or foam impression box techniques, with a typical unit and supporting software costing from €6-10K and systems suitable for producing foot and lower leg scans that can be used to generate ankle-foot orthotics over twice that. In addition, the company who manufacture the orthotics need to have the manufacturing facilities in place to fabricate the orthotic from the computer model of the mould, usually done using CNC milling. These manufacturing equipment costs are offset somewhat by the equipment required for manufacturing from a traditional plaster cast, which requires vacuum pumps, grinders and other workshop equipment.

It is worth noting that at the moment 3D scanning technologies are limited as to how much adjustment to the foot the clinician can make while it is being scanned. It has been previously noted in the literature however that, depending on the clinician, there is a significant variation in the position the foot is cast in using traditional methods [41].

### Dynamic measurements

Attempts have been made to use optical technologies to measure dynamic changes in foot shape during normal walking [10,42,43]. The equipment setup required to produce this data however remains relatively complex and expensive, for example the methodology used by Kimura et al [10] required a purpose built raised walkway and 12 video cameras to carry out their study. Further research will undoubtedly reduce cost and equipment requirements but it has yet to be shown that the capture of 3D movement of the foot would provide any clinically relevant insight into a subject's foot function beyond that which is currently possible to analyse using standard motion capture systems.

### Foot assessment

Beyond their role in the prescription of foot orthotics and customised shoes intended to accommodate deformities, there is some potential to use 3D scanning technologies for research and clinical assessments of medical conditions relating to the foot. Borchers et al [44] were the first to investigate a laser scanner with a view to assessing its potential for informing the design of shoes intended to reduce the risk of ulceration in insensate feet. Limitations with the technology at the time meant that the authors had difficulties in aligning parts of the foot scan but they were able to show that, compared to a standard shoe last, the hallux and the 5^th ^metatarsal head both protruded outside of the last shape, both areas which are common ulceration sites for diabetic patients.

Chen et al [8] used a 3D scanner to measure forefoot varus angle in individuals with flexible flatfoot and found it to be a "fast and accurate" measurement technique. Scans were taken of a cast of the foot rather than directly, and subjects with flatfoot were found to have a varus angle 3.6° greater than those in the control group. Although the study found significant differences between the groups they did not compare the results to those which would have been found using a standard clinical approach. A more recent study using 3D scanning to investigate flatfoot prevalence in a group of 835 children was carried out by Pfeiffer et al [45], and found that age, gender and weight were the key influences on flat foot development. The Infoot 3D foot digitiser (I-Ware Laboratory Co., Ltd, Osaka, Japan), the development of which has been described by Kouchi and Mochimaru [46], has been investigated for validity and reliability compared to manual measurements for rheumatoid arthritis patients [9]. The device was found to be reliable with high intraclass correlation coefficients for linear values, although standard errors of measurement were found to be up to 5.9 mm for girth measurements. The authors concluded that the device was a fast and reliable method of obtaining 3D anthropometric data of the foot.

### Other 3D foot scanning research

A number of additional studies have been carried out related to 3D foot scanning, sometimes using innovative methods [47,48]. Martedi and Saito [47] recently reported on their attempts to use a standard flatbed scanner - the type that would normally be found in the office environment for digitising documents - to scan the foot sole and translate the output to a 3D form. The distance of the sole away from the scanner glass was estimated using the albedo of the sole surface and the pixel intensity of the resulting image, inspired by techniques used in the analysis of satellite images. The authors claim they are able to achieve an average error of <1 mm, in line with those achieved by more expensive scanning systems, however the system was tested using a foot model with a uniform colour and it was noted that scanning a real foot, especially those with damage or injury could present problems for the reconstruction process.

Witana et al [49] used 3D scanning to assess the foot shape deformation of 16 subjects whilst standing on foot supports made out of different materials. Markers were attached to the foot on several bony landmarks and it was the changing position of these that were used to measure foot deformation. This approach was able to show significant differences in foot shape depending on the surface material used and this could potentially have applications for the prescription of orthotics.

In a recent study, Mauch et al [50] scanned the feet of almost 3,000 children and identified 5 foot types: flat, robust, slender, short and long. By looking at the distribution of foot types in normal, overweight and underweight children the data generated were able to show a higher prevalence of flat and robust feet in overweight children, and slender and long feet in those that were underweight for their age.

Measuring the surface area of the foot is another application of 3D scanning. This area has been traditionally estimated as percentage of the total body surface area [51], or as a formula based on linear foot measurements [52]. By using a scanning system it is possible to increase the accuracy of the measurement by taking into account many parts of the foot surface that are missed using previous, physical measuring techniques such as wrapping [53].

## Conclusion and future recommendations

There are a number of current and potential applications for 3D scanners in commercial, clinical and research areas related to the human foot. While there may be improvements that could be made with regards to software designed to automatically take measurements from foot scans, it has been shown that the 3D scans produced by these systems are accurate representations of the foot and that the measurements taken from them are in general comparable to those that would be taken manually. The foot scanner's role in orthosis and customised shoe design and manufacture has been established, where it provides time and cost advantages over traditional casting techniques in return for a greater initial outlay. Initial research suggests that foot orthotics designed from 3D scans of the foot are at least comparable with those made through traditional methods, although further research is required to confirm this.

The utility of scanning systems for clinical and research purposes has been successfully demonstrated, particularly for anthropometric measurement. 3D scanners allow large numbers of subjects to be scanned quickly and easily, with the data available for analysis at a convenient time for the researcher. There would appear to be scope for the expansion of scanner-based research into the investigation of a range of foot conditions, for example those that require the monitoring of the progression of a deformity over time. This approach could help to reduce radiation exposure to the patient from x-rays.

The use of 3D scanning technologies to gain a better understanding of the changes in the shape of the foot under different loading conditions that relate to the conditions it will be under during normal use - walking, going up stairs for example - has been investigated and could be an application relevant for the design of footwear and orthotic devices. For example, the measurement of the change in arch height under different loading can be used to inform the design of the orthotic, and while this can currently be achieved using kinematic analysis of motion capture data from gait labs, the majority of podiatrists and other clinicians who prescribe orthoses do not have these facilities. A small and reasonably priced scanning unit combined with software that can quickly analyse the changes and provide advice for the prescription could be beneficial in this situation. Producing actual dynamic 3D scans of the foot during gait has been achieved, however the quality of these scans and complexity of the equipment setup required to make them means that this option is several years from being commercially available at a clinical level.

Studies have demonstrated some variation in the results obtained from 3D scans using different techniques. Ideally, to maximise time and cost savings the scan should be taken directly of the foot to remove the need for casting. In order to make it a standard component of an assessment for a foot orthotic or customised shoe it is essential that a standardised protocol is developed describing the preparation of the foot (for example cleaning, elevating beforehand), processing of the scan data, and, if required, points where measurements should be taken from. ISO 7250 states that measurements of foot length and width should be taken with the subject in a standing position, 50% of their weight on each foot. However it is not clear from the current evidence bases what is the best level of weight bearing that will give the best quality shoes or orthotics. Current standards are also limited to linear measurements of the foot, and it is suggested that these should be expanded to include relevant girth measurements so that these can be included in anthropometric databases. Identifying bony landmarks on the foot using markers that show up on the 3D scan appears to be the most reliable method of obtaining accurate girth measurements.

Bringing researchers in the field, scanning equipment manufacturers, orthotic and footwear companies, end users and other stakeholders together to further explore these issues may result in cross disciplinary activity needed to resolve current needs and issues.

## Competing interests

The authors declare that they have no competing interests.

## Authors' contributions

ST and JW conceived the initial idea for the review. ST carried out the initial literature searches. Both authors drafted and prepared the manuscript and approved the final manuscript.
